# Insecticidal Properties of a Highly Potent Wax Isolated from *Dolichandra cynanchoides* Cham

**DOI:** 10.3390/molecules21081039

**Published:** 2016-08-11

**Authors:** Georgina Díaz Napal, María C. Carpinella, Sara M. Palacios

**Affiliations:** Unidad Asociada Area Cs. Agr. Ing. Bio. y S.-CONICET-Universidad Católica de Córdoba, Armada Argentina 3555, 5016 Córdoba, Argentina; georgidnapal@hotmail.com (G.D.N.); ceciliacarpinella@ucc.edu.ar (M.C.C.)

**Keywords:** *Dolichandra cynanchoides*, wax, *Spodoptera frugiperda*, *Epilachna paenulata*, antifeedant

## Abstract

Bioassay-guided fractionation of an ethanolic extract of the aerial parts of *Dolichandra cynanchoides* Cham. (Bignoniaceae) led to the isolation of a natural wax with anti-insect activity against *Spodoptera frugiperda* (Noctuidae) and *Epilachna paenulata* (Coleptera). The compound was identified spectroscopically as an ester of a C27 fatty acid and a C25 alcohol, pentacosyl heptacosanoate (**1**). The effective doses of **1** for 50% feeding inhibition (ED_50_) of *S. frugiperda* and *E. paenulata* were 0.82 and 8.53 µg/cm^2^, respectively, in a choice test, while azadirachtin showed ED_50_ of 0.10 and 0.59 µg/cm^2^, respectively. In a no-choice test, both insects refused to feed on leaves treated with **1** at doses of 0.1 µg/cm^2^ or greater inhibiting larval growth and dramatically reducing survival. The lethal doses 50 (LD_50_) of **1** were 0.39 and 0.68 µg/cm^2^ for *S. frugiperda* and *E. paenulata*, respectively. These results indicate that **1** has potential for development as botanical insecticides. Similar esters might be obtainable in large quantities as many edible crops produce wax esters that are discarded during food processing. Research on these materials could lead to the detection of similar waxes with insecticidal activity.

## 1. Introduction

To defend themselves, plants synthesize a range of secondary metabolites, which provide resistance against phytophagous insects [1], and may affect various physiological processes of insects through antifeedant, toxic and growth-regulating activity [2].

We have screened >100 native plants from central Argentina for natural pesticides [3,4], and found that *Dolichandra cynanchoides* Cham. showed potent antifeedant activity against the Coleoptera *Epilachna paenulata. D. cynanchoides* is a vine belonging to the Bignoniaceae family, native to the Chaco region of South America and densely distributed from north to central Argentina. Its aerial parts were used by native peoples as a medicine for the treatment of diarrhea and nausea and also used for handcrafts [5]. Ethanolic leaf extracts from *D. cynanchoides* acted as an antifeedant against Coleoptera, as a deterrent against aphid settling, but is benign against honeybees, a beneficial insect [6].

A sustainable approach to pest control requires the discovery of novel compounds that affect the behavior and survival of insects and that can replace synthetic insecticides. Consequently, we focused on identifying the active principle of *D. cynanchoides* using a generalist (*Spodoptera frugiperda*) and a specialist (*E. paenulata*) insect for the bioguided isolation and characterization of its insecticidal action.

## 2. Results and Discussion

Bioguided separation of components of the ethanolic extract of the aerial parts of *D. cynanchoides* led to the isolation of a wax, identified as pentacosyl heptacosanoate (**1**), an ester formed by a fatty acid and a fatty alcohol, as its active principle. Recovery was 0.6 g/100 g of dried plant material.

**Figure 1:**
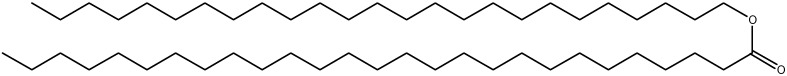
Chemical structure of pentacosyl heptacosanoate (**1**).

Compound **1**, obtained as a colorless solid, was analyzed spectroscopically. Its IR spectrum showed absorption at 1732 and 1281 cm^−1^ for the ester, and 2919 and 2853 cm^−1^ for the long chain. In its ^1^H-NMR spectrum, the oxymethylene moiety was deduced from a triplet at 3.64 ppm (*J* = 6.6 Hz) and a signal at 63.13 ppm in the ^13^C-NMR. A methylene adjacent to the carbonyl group was observed at 2.35 ppm as a triplet (*J* = 7.4 Hz) in the ^1^H-NMR spectrum and the carbon signal of this methylene group appeared at 32.82 ppm in ^13^C-NMR. Proton signals of two methylenes were observed at 1.63 ppm and 1.57 ppm as multiplets, adjacent to the oxymethylene and to the carboxymethylene, respectively, according to the COSY experiment. From the HSQC experiment, it was deduced that carbon signals at 31.99 ppm and 29.71 ppm corresponded to these methylenes, respectively. The carbonyl carbon signal appeared at 178.13 ppm. The protons of two terminal methyl groups were observed as a broad triplet at 0.88 ppm and the corresponding carbons appeared at 14.13 ppm in the ^13^C-NMR spectrum. A DEPT experiment showed that no tertiary carbons were present, ruling out any possibility of branching. It was thus clear that compound **1** was an ester composed of a linear long chain alcohol and a fatty acid. It contained 104 protons as calculated from ^1^H-NMR and also as established by ESI-Q-TOF. The molecular formula was determined as C_52_H_104_O_2_ (*m/z* = 761.3829) and the acid moiety, confirmed by its MS fragmentation pattern, was characteristic of an ester with intense peaks at *m/z* = 393 and at *m/z* = 365 of the cleavage of the alkyl moiety, indicating the acid moiety as C_27_H_53_O_2_ and the alkyl moiety as C_25_H_51_. The structure was thus confirmed as pentacosyl heptacosanoate (**1**) (Figure 1). 

The wax that plants produce is mainly deposited on the cuticle, the outer surface of the epidermal cells of all plant parts. The cuticle prevents dehydration and provides protection from environmental stresses such as UV radiation, pathogens, and insects [7]. Chemically, plant waxes are a mixture of fatty esters, *n*-alkanes, fatty alcohols and free fatty acids of different chain lengths [8]. When we extracted intact leaves of *D. cynanchoides* with hexane for three minutes, we found higher yields of **1** (3.7 g/100 g plant material), suggesting that this compound is part of the cuticular waxes of this plant.

### 2.1. Choice Assay against S. frugiperda and E. paenulata

Compound **1** showed antifeedant activity against the generalist insect, *S. frugiperda*, and the specialist insect, *E. paenulata*, with the ED_50_ (0.82 µg/cm^2^) for the polyphagous *S. frugiperda* being ten times higher than that for the specialist *E. paenulata* (ED_50_ = 8.53 µg/cm^2^) (Table 1). We compared the ED_50_ of **1** with that of azadirachtin (**2**), and found an eight-fold difference for *S. frugiperda* (Table 1), but a 14-fold difference in *E. paenulata*. As far as we know, this is the first report about antifeedant activity exerted by **1**. There are many reports that alkanes in particular have harmful effects on insects [2], but little is known about the effect of wax esters. Other structurally related compounds are bioactive against different species of insects. For example, octanoic acid (C_8_) was toxic against *Drosophila* *melanogaster*, but inactive against *D. sechellia* [9]. Fatty acids, such as oleic, linoleic, and linolenic acids, deterred the settling of *Myzus persicae* and *Rhopalosiphum padi* [10]. Pentacosanol (C25 alcohol) was a deterrent against *Plutella xylostella* larvae [11] and triacontanol (C30 alcohol) exhibited antifeedant activity against *S. litura* with an ED_50_ of 5.42 µg/cm^2^ [2,12].

Although many iridoid glycoside compounds have been isolated from *D. cynanchoides* [13,14] and iridoid glycosides have been reported as deterring consumption by generalist insect herbivores [15], we did not find iridoids to be among the most active antifeedant compounds. Compound **1** did show strong antifeedant activity, suggesting that *D. cynanchoides* mostly relies on **1** for its defense against herbivores.

### 2.2. No-Choice and Mortality Assays against S. frugiperda

**Figure 2:**
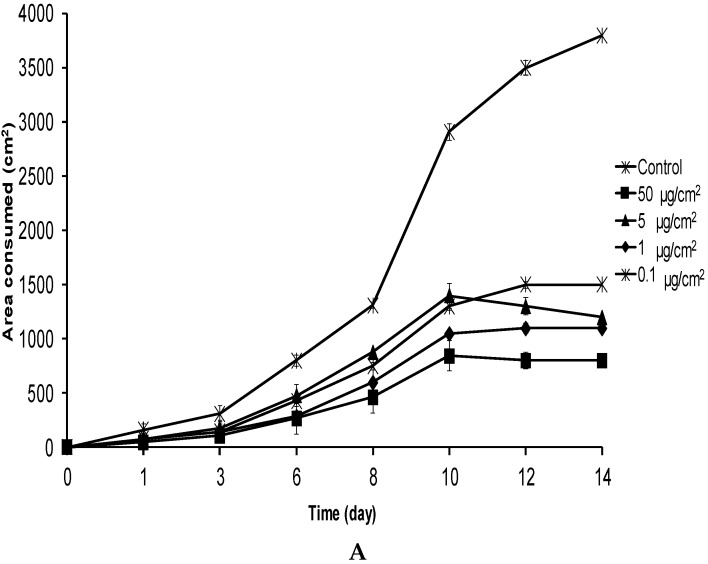

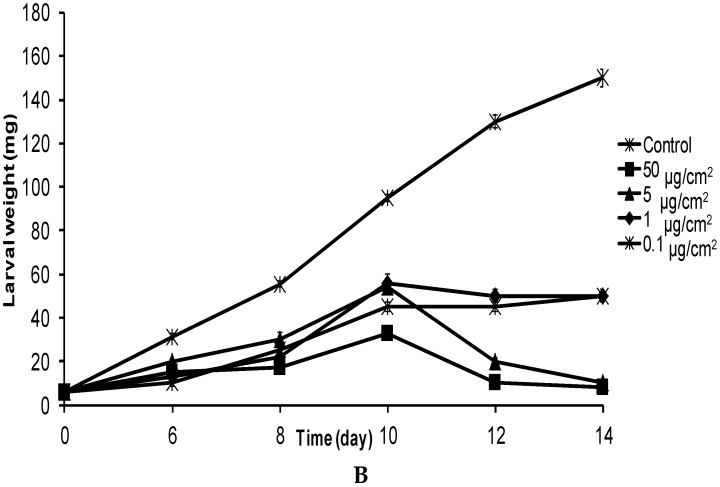
Average leaf area consumed (**A**) and average body weight (**B**) of *S. frugiperda* larvae fed on leaves treated with **1** in a no-choice feeding assay. Error bars <60 and <10 for area consumed and body weight, respectively, are embedded in symbols.

*S. frugiperda* larvae were fed on leaves treated with **1** at 0.1–50 µg/cm^2^, and at six days, they were strongly deterred, consuming up to three times less food than the controls (Figure 2A) with the observation of some mortality (20%–40%). After 10 days of exposure, larvae receiving **1** at 1, 5, and 50 µg/cm^2^ were not feeding at all, and larvae treated at 0.1 µg/cm^2^ consumed significantly less food than controls (*H* = 5.07; *p* = 0.019). This behavior was maintained until the end of the experiment (d14) when all the individuals were in pupae in the control, while in the treatments of 50, 5, 1 and 0.1 µg/cm^2^, all the remaining larvae (two, two, four, and three individuals, respectively) were in instar LII–III. The effect of **1** at 0.1 µg/cm^2^ was remarkable because few natural compounds are effective at this concentration [16], and even more so considering that the molar concentration of **1** was equivalent to 130 pmol/cm^2^.

This effect was clearly reflected in insect weight gain. While control larvae body weight steadily increased, the body weight of all larvae fed on leaves treated with **1** at 0.1–50 µg/cm^2^ was highest at 10 days after treatment and then decreased, probably due to starvation (Figure 2B). The initial average weight of control larvae was 6 ± 0.1 mg, reaching 150 ± 18 mg at day 14. In the case of larvae treated with 50 µg/cm^2^ of **1**, the average weight was 6 ± 0.1 mg at the beginning and only 10 ± 2.2 mg at 14 days after initiation of the experiment. The larval weights for insects treated with the 5, 1, and 0.1 µg/cm^2^ concentrations at 14 days were 8 ± 2, 46 ± 5, and 45 ± 5 mg, respectively, reflecting the dose-dependent, high impact of **1** in this insect. 

**Figure 3:**
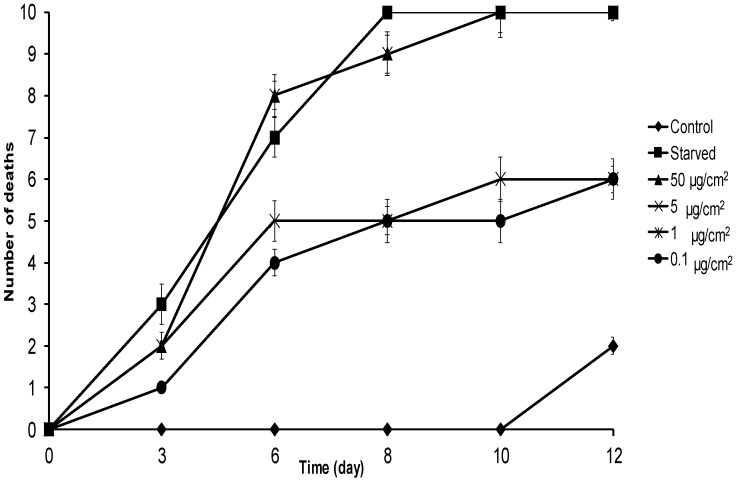
Cumulative mortality of *S. frugiperda* larvae fed with leaves treated with **1** at different concentrations. All treatments were in triplicate (*n* = 10). Starved represents food-deprived treatment. Error bars show standard deviation; where error is not shown, it is smaller than the symbol.

In the mortality assay, all larvae treated with **1** at 50 µg/cm^2^ died 10 days after the beginning of the assay, while all food-deprived larvae died by day 8. Doses of 0.1 and 1 µg/cm^2^ showed 50% mortality at day 12 (Figure 3). The LD_50_ of **1** at eight days was 0.39 (0.05–2.5) µg/cm^2^, while lethal times 50 (LT_50_) for **1** (Table 2) were 3.14 and 3.31 days for the 50 and 5 µg/cm^2^ treated groups, respectively. The LT_50_ for food-deprived larvae was 3.15 days. These results suggest that **1** strongly affects the food intake of *S. frugiperda*, consequently disrupting insect development and provoking low body weight and high mortality.

Waxes play an important role in plant–insect interactions, adversely affecting insects through direct toxicity or by hindering their physical movement [17]. The latter was extensively studied [18], but few reports were found for the former. Yang et al. found that five wild peanut species were more resistant to *S. frugiperda* larvae in feeding bioassays than a peanut cultivar. Larvae reared on foliage of the wild peanut species showed 79%–91% mortality compared to 17% for the cultivated peanut [19]. When they removed the epicuticular lipids from the foliage of wild and cultivated peanut species, *S. frugiperda* exhibited increased larval weights and earlier pupation and adult emergence than insects reared on a diet with untreated foliage, indicating that it was the wax mixture that negatively affected insect survival.

In a similar study, *S. frugiperda* larvae grew better when they were fed a diet containing corn foliage with the epicuticular lipids extracted than one with unextracted foliage [20], but diets incorporating lipid extracts containing no or trace amounts of wax esters had no effect on the insects [20]. Some resistant alfalfa plants (*Medicago sativa* L.) were found to have leaf surfaces with as much as 50% more wax esters than plants that are susceptible to spotted alfalfa aphids (*Therioaphis maculata*) [21], suggesting that wax esters are involved in this preference. In spite of many reports about the role of wax and wax esters and their effects against insects, we found no reports of the antifeedant action of the ester isolated here or similar ones. 

### 2.3. No-Choice and Mortality Assays against E. paenulata

**Figure 4:**
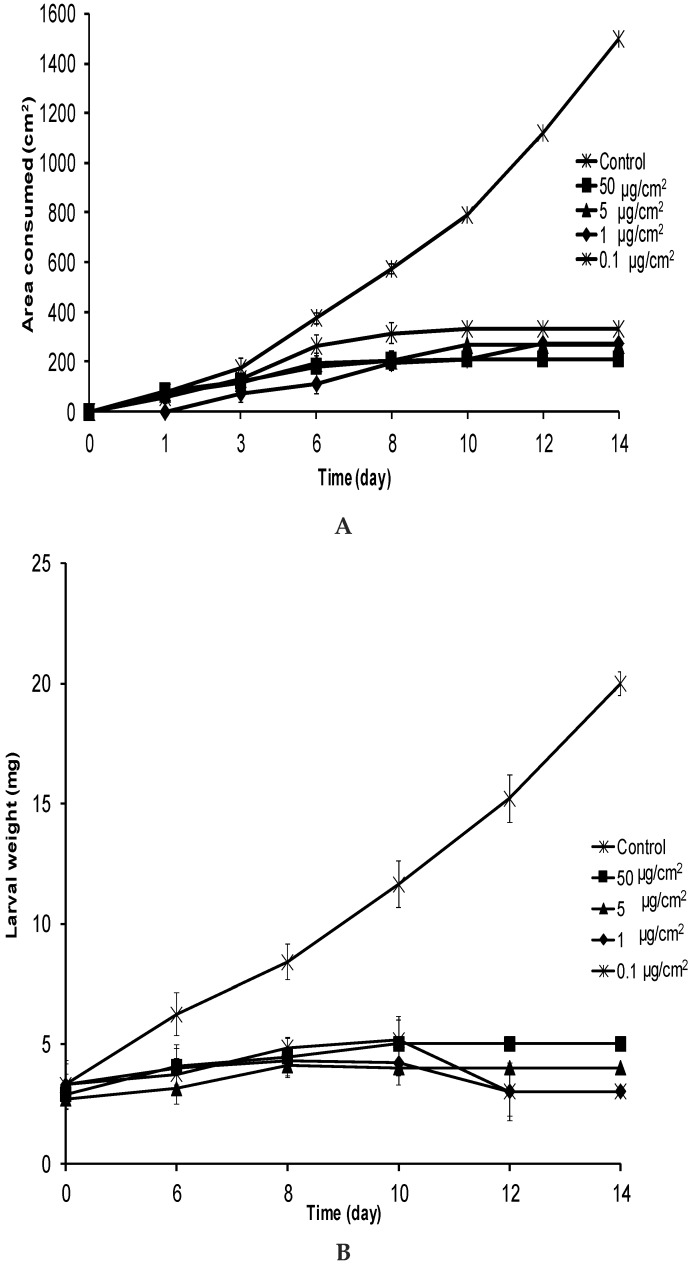
Average leaf area consumed (**A**) and average body weight (**B**) of *E. paenulata* larvae fed on leaves treated with **1** in a no-choice feeding assay. Error bars <30 and <2 for area consumed and body weight, respectively, are embedded in symbols.

After 3 days, *E. paenulata* larvae significantly reduced their intake of food treated with **1** at 0.1, 1, 5, or 50 µg/cm^2^ (*p* < 0.05) (Figure 4A). After six days of exposure to **1** at 1, 5, and 50 µg/cm^2^, *E. paenulata* larvae had consumed less than half the amount of leaf material as larvae facing untreated leaves (*H* = 21.87; *p* < 0.001) (Figure 4A). These differences increased with time and, after 12 days of exposure, larvae receiving **1** at all concentrations were not feeding at all, with four, three, three and two remaining individuals at 0.1, 1, 5, and 50 µg/cm^2^, respectively. The presence of **1** on insect food clearly led to larval starvation. *E. paenulata* larvae exposed to **1** at all concentrations did not gain weight (Figure 4B), whereas the body weights of the control larvae continually increased. At day 6, there were significant differences in body weight (*H* = 18.85; *p* < 0.001) between control larvae and those receiving food treated with **1** at any concentration (Figure 4B). After 14 days, larval weight had declined to less than the initial body weight.

**Figure 5:**
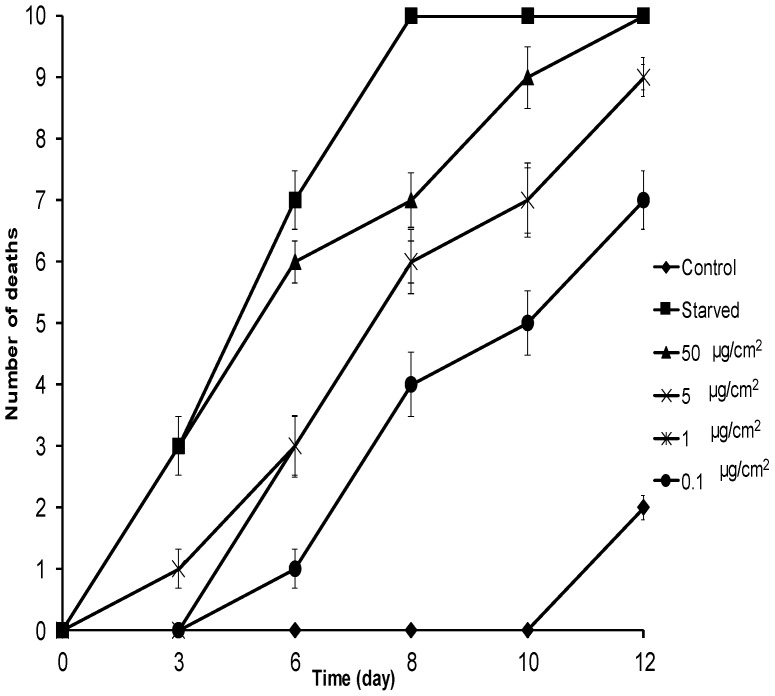
Cumulative mortality of *E. paenulata* larvae fed with leaves treated with **1** at different concentrations. All treatments were in triplicate (*n* = 10). Starved represents food-deprived treatment. Error bars show standard deviation; where error is not shown, it is smaller than the symbol.

In the mortality assay, after six days, the mortality rates of *E. paenulata* larvae receiving treated food were significantly higher than those of controls (*H* = 14.21; *p* = 0.004) for all concentrations including 0.1 µg/cm^2^ (Figure 5). Leaf treatment with 50 µg/cm^2^ of **1** induced 100% mortality after 12 days (Figure 5). With 5 µg/cm^2^ and 1 µg/cm^2^, mortality was 90%, and with 0.1 µg/cm^2^, it was 60% at the same time point. Food-deprived larvae did not survive longer than eight days. The LD_50_ of **1** at eight days was 0.68 µg/cm^2^ (0.01–1.99). Comparing this value with that reported for **2** (LD_50_ =1.24 µg/cm^2^) at four days [22] for the same insect suggests that **1** is as toxic as **2**, one of the strongest known natural insect toxicants. Likewise, the toxicity of **1** against *E. paenulata* was similar to that of the limonoid meliartenin (LD_50_ = 0.76 µg/cm^2^ at 4 days) [22], another very strong natural insecticide. It was also more potent than other plant-derived compounds such as the flavonoid pinocembrin (LD_50_ = 18.4 µg/cm^2^ at six days), quercetin (LD_50_ > 50 µg/cm^2^ at 12 days) [23], and the sesquiterpene artemisinin (LD_50_ ~30 µg/cm^2^ at seven days) [24]. Lethal times 50 (LT_50_) for **1** (Table 2) was 4.69 days for 50 µg/cm^2^ compared to 4.10 days for food-deprived larvae, suggesting that larvae fed on leaves treated with **1** died from starvation. The feeding deterrent effects of **1** against *E. paenulata* larvae had strong consequences for their performance and survival.

Wax esters are biodegradable, non-toxic, and are obtained from renewable sources [25] including vegetable oil crops such as olive [26], soya [27] and sunflower [28]. Similar esters of different chain lengths may also act as insecticides. Consequently, our results encourage the study of fatty esters from crop residues, such as leaves and seeds, from vegetable oil processing waste, etc., in order to find new sources of potent anti-insect compounds.

## 3. Materials and Methods

### 3.1. Plant Material

Aerial parts of *D. cynanchoides* were collected in the Calamuchita Valley, Córdoba, Argentina (31°68′ S 64°43′ W) in March 2013. A voucher specimen (UCCOR 120) has been deposited at the Herbarium Marcelino Sayago of the School of Agricultural Sciences, Catholic University of Córdoba, and was identified by the agronomist Gustavo Ruiz. Air-dried aerial parts of *D. cynanchoides* (602 g) were extracted with ethanol for 96 h at room temperature. After removal of the solvent (reduced pressure), an extract was obtained (45.60 g, 7.57% yield).

### 3.2. General Experimental Procedures and Apparatus

1D- and 2D-NMR spectra were recorded in CDCl_3_ with a Bruker AVANCE II 400 spectrometer (Bruker, Billerica, MA, USA) operated at 400 MHz for ^1^H and at 100 MHz for the ^13^C nucleus. Tetramethylsilane (TMS) was used as the internal reference (δ 0.00). The high resolution mass spectra were determined in a Bruker MicroQTOF-II mass spectrometer (Bruker), equipped with an ESI source operated in positive mode at 180 °C with a capillary voltage of 4500 V. Mass accuracy was verified by calibration before and after sample introduction, using sodium formate (1 mM). Both samples and calibrant were introduced using a syringe pump at 10 µL min^−1^. Electron impact mass spectra (EI-MS) were obtained at 70 eV by GC–MS on a Hewlett–Packard 5970 Series mass spectrometer interfaced (Hewlwtt-Packard, Palo Alto, CA, USA) with a Hewlett–Packard 5890 gas chromatograph fitted with a column (HP-5MS, 15 m × 0.25 mm i.d., temperature from 200–290 °C, 10 °C/min). HPLC was performed in a Shimadzu chromatograph with a diode array UV-Vis detector (Shimadzu, Kyoto, Japan). Fourier transform infrared spectra were acquired on a FTIR Nicolet 510P spectrometer (Thermo Scientific, Waltham, MA, USA). Spectra over a range of 4000–500 cm^−1^ with a resolution of 2 cm^−1^ (50 scans) were recorded using KBr pellets.

### 3.3. Chemicals and Chromatographic Absorbents

Analytical TLC was performed on silica gel 60 F_254_ Merck plates (Darmstadt, Germany). Silica gel grade 200–400 mesh, 60 Å, for column chromatography was purchased from Sigma Chemical Co., Inc. (St. Louis, MO, USA). All solvents were purchased from Merck and Fisher Scientific (Buenos Aires, Argentina). Azadirachtin (**2**) (95% purity) was purchased from Sigma-Aldrich Argentina (Buenos Aires, Argentina).

### 3.4. Insects

*S*. *frugiperda* and *E. paenulata* larvae were obtained from a laboratory colonies. *E. paenulata* was reared on a natural diet of *Curcubita maxima* leaves [23] while *S. frugiperda* was reared on an artificial diet [29]. Both insects were maintained in a growth chamber at 24 ± 1 °C and 70%–75% relative humidity with a photoperiod of 16/8 h light-dark cycle, and periodically renewed with field specimens.

### 3.5. Feeding Choice Assay

The antifeedant experiments were carried out by the leaf-disk choice test for compounds **1** and **2** [30]. Two circular sections of the leaves of *Lactuca sativa* or *C. maxima* for *S. frugiperda* and *E. paenulata*, respectively, were placed in a Petri dish and a glass disk with two 1 cm^2^ holes was placed on top. One disk was treated with 10 µL of test solution and the other with 10 µL of Et_2_O (solvent control), and then both pieces were allowed to stand for 15 min until the solvent was completely evaporated. A third-instar larva of the corresponding insect was placed equidistant from the treated and untreated leaf disks and allowed to feed for 3 h or 24 h for *S. frugiperda* or *E. paenulata* larvae, respectively. Test solutions were prepared dissolving the necessary amount of **1** or **2** (reference of a potent natural antifeedant compound) in 10 mL of analytical grade Et_2_O. The dosages applied on the leaf offered for each compound were 0.1, 1, 5 and 50 µg/cm^2^. Ten replicates were conducted for each treatment. The relative amounts (recorded in percentages from 0 to 100) of the treated and untreated substrate area eaten in each test were estimated visually by dividing the food area into imaginary quarters or by determining the area of the leaf disks consumed by the larvae using the ImageJ screener software program (Fiji project. Available online: http://imagej.net/Downloads). An antifeedant index (AI%) was calculated as [(C − T)/(C + T)] × 100 [31], where T and C represent consumption of treated and untreated foods, respectively. Then, the ED_50_ (effective dose to obtain 50% feeding inhibition) was determined with the AI% values, from the Probit regression.

### 3.6. No-Choice Feeding Assay

One *S. frugiperda* or *E. paenulata* first instar larva was placed in a Petri dish and fed on *L. sativa* or *C. maxima* leaves, respectively, on which aliquots of 0.1, 1, 5 and 50 μg/cm^2^ (10 µL) of either **1** or Et_2_O (control, 10 µL) were applied with a Hamilton syringe. The leaves were renewed every 24 h. Ten replicates were conducted for each treatment and each trial was repeated twice. Leaf consumption and body weight were recorded every 24 h and 72 h, respectively [23].

### 3.7. Mortality Assay

Groups of 10 *S. frugiperda* or *E. paenulata* larvae (first instar) were continuously fed on leaves treated with either 0.1, 1, 5 or 50 μg/cm^2^ of **1** or with solvent (Et_2_O 10 µL) as control. A similar set of larvae was not fed at all and acted as food-deprived controls. Three replicates were performed for each treatment. Mortality was recorded every 24 h. Lethal dose LD_50_ and lethal time LT_50_ values for **1** were determined from mortality data by Probit analysis. 

### 3.8. Isolation of Pentacosyl Heptacosanoate *(**1**)*

The extract of *D. cynanchoides* (3.50 g) was partitioned between Et_2_O/H_2_O. The Et_2_O soluble extract (0.87 g) showed AI% = 100, while the aqueous soluble fraction (2.53 g) was inactive. The organic fraction was fractionated by silica column chromatography and eluted with a gradient of hexane/Et_2_O and MeOH, obtaining 33 fractions according the TLC (eluted with hexane/Et_2_O (80:20)). The active fractions (F^1^ 6–10) (AI% = 90% at 50 µg/cm^2^) were re-chromatographed on silica gel with hexane/Et_2_O 80:20, yielding 10 fractions. The most active fraction, F^2^ 4 (AI% = 94%), presented a major component that precipitated as a solid. This compound was isolated (TLC with toluene/Et_2_O (70:30) Rf = 0.38), recrystallized from Et_2_O, and assayed against *E. paenulata*, showing AI% = 100% at 50 µg/cm^2^. The purity of this compound was determined by HPLC performed on a Kromasil direct-phase column (4.6 mm i.d. × 250 mm) (Akzo Nobel, Pasadena, CA, USA), eluting with hexane–isopropanol 95:5 as the mobile phase and UV detection at 210 nm. This methodology was also used to determine the yield of the active compound in plant material. The compound was identified as pentacosyl heptacosanoate (**1**) (Figure 1) by ^1^H-NMR, ^13^C-NMR, MS, and IR spectroscopies.

Pentacosyl heptacosanoate (**1**) white solid, IR, 2919, 2653, 1732, 1730, 1454, 1374, 1281, 1069 cm^−1^. ^1^H-NMR (CDCl_3_, 400 MHz) δ 0.88 (6H, t, *J* = 6.8 Hz, 2 × CH_3_) 1.25 (90H, 45 × CH_2_), 1.57 (2H, m; CH_2_), 1.63 (2H, m; CH_2_), 2.35 (2H, t, *J* = 7.4, CH_2_-CO), 3.64 (2H, t, *J* = 6.6, CH_2_-O); ^13^C-NMR (CDCl_3_, 100 MHz) δ 14.13 (CH_3_), 22.71 (CH_2_), 25.75 (CH_2_), 29.71 (CH_2_), 31.94 (CH_2_), 32.82 (CH_2_-CO), 63.13 (CH_2_-O), 178.13 (C=O). ESI-Q-TOF MS: *m*/*z* 761.3829 (calcd. for C_52_H_104_O_2_, 761.3823, error 0.78 ppm). EIMS: *m*/*z* 393 (C_27_H_53_O), 365 (C_26_H_53_), 351 (C_25_H_51_), 111, 97, 83, 71, 57.

### 3.9. Statistical Analysis

Results from choice tests were analyzed by the Wilcoxon Signed Rank Test. Data were analyzed for normal distribution and homoscedasticity and, as they did not meet these requirements, non-parametric tests were used. Results on average larval body weight, accumulated consumption values from no-choice tests, and mortality from the mortality assay were compared with respect to concentrations by the Kruskal–Wallis non-parametric analysis of variance, followed by the Dunn test. Statistical analyses were performed using the InfoStat statistical package (version 2008, UNC, Córdoba, Argentina). Differences were considered significant at *p* ≤ 0.05.

## 4. Conclusions

The antifeedant effect of **1** on generalist and specialist insects, in both the choice and no-choice tests, showed negative consequences for insect survival, suggesting that the use of this compound is worth evaluating as an insecticide. This wax, with its simple chemical structure that is easy to synthesize or mimic, may even be considered as a model for designing new eco-friendly insecticides. In addition, many edible crops produce wax esters that are discarded during processing for food. Research on these materials could lead to the detection of similar waxes with insecticidal activity.

## Figures and Tables

**Table 1:** Effective antifeedant dose 50 (ED_50_) of **1** and **2** against *Spodoptera frugiperda* and *Epilachna paenulata* determined by choice test.

Compound	Insect	ED_50_ (µg/cm^2^) (Values and 95% Confidence Interval)
Pentacosyl heptacosanoate (**1**)	*S. frugiperda*	0.82 (0.15–4.50)
Pentacosyl heptacosanoate (**1**)	*E. paenulata*	8.53 (2.73–26.59)
Azadirachtin (**2**)	*S. frugiperda*	0.10 (0.05–0.19)
Azadirachtin (**2**)	*E. paenulata*	0.59 (0.07–4.37)

**Table 2:** Lethal time (LT_50_) of **1** against *Spodoptera frugiperda* and *Epilachna paenulata* in mortality test.

Dosage µg/cm^2^	LT_50_ in Days (Values and 95% Confidence Interval) ^a^	
	1	Food-Deprived Larvae
*Spodoptera frugiperda*		
50	3.14 (2.88–6.40)	
5	3.31 (3.10–6.70)	
1	6.96 (3.62–16.00)	
0.1	7.71 (4.81–16.53)	
		3.16 (2.63–6.03)
*Epilachna paenulata*		
50	4.69 (2.78–7.80)	
5	7.56 (5.62–9.96)	
1	7.80 (4.89–10.06)	
0.1	9.62 (7.06–13.13)	
		4.10 (2.59–6.19)

^a^ LT_50_ is the time required to obtain 50% mortality. LT_50_ was calculated by Probit log regression.
